# Changes in COVID-19 Vaccine Hesitancy Among Black and White Individuals in the US

**DOI:** 10.1001/jamanetworkopen.2021.44470

**Published:** 2022-01-21

**Authors:** Tasleem J. Padamsee, Robert M. Bond, Graham N. Dixon, Shelly R. Hovick, Kilhoe Na, Erik C. Nisbet, Duane T. Wegener, R. Kelly Garrett

**Affiliations:** 1 College of Public Health, The Ohio State University, Columbus; 2 School of Communication, The Ohio State University, Columbus; 3 Department of Communication and Media, Merrimack College, North Andover, Massachusetts; 4 School of Communication, Northwestern University, Evanston, Illinois; 5 Psychology Department, The Ohio State University, Columbus

## Abstract

**Importance:**

COVID-19 has disproportionately affected Black individuals in the US; however, vaccination rates among Black individuals trail those among other racial groups. This disparity is often attributed to a high level of vaccine hesitancy among Black individuals, but few studies have examined changes in vaccine hesitancy over time.

**Objectives:**

To compare changes in vaccine hesitancy between Black and White individuals in the US and to examine mechanisms that might help explain the observed differences.

**Design, Setting, and Participants:**

This survey study used 7 waves of data collected using a panel design. A total of 1200 English-speaking adults in the US were recruited from a nonprobability online panel to construct a census-matched sample. Participants were contacted monthly between December 9, 2020, and June 16, 2021.

**Main Outcomes and Measures:**

The main outcome of interest was self-reported vaccination intention, measured on a 6-point scale (where 1 indicates extremely unlikely and 6 indicates extremely likely). Beliefs about the safety, effectiveness, and necessity of COVID-19 vaccines were measured on a 5-point Likert scale, with higher scores denoting greater agreement.

**Results:**

The baseline data included 1200 participants (693 women [52.0%; weighted]; 921 White individuals [64.0%; weighted], 107 Black individuals [12.2%; weighted]; weighted mean [SD] age, 49.5 [17.6] years). The survey participation rate was 57.0% (1264 of 2218). Black and White individuals had comparable vaccination intentions in December 2020, but Black individuals experienced larger increases in vaccination intention than White individuals relative to baseline in March 2021 (*b* = 0.666; *P* < .001), April 2021 (*b* = 0.890; *P* < .001), May 2021 (*b* = 0.695; *P* < .001), and June 2021 (*b* = 0.709; *P* < .001). The belief that the vaccines are necessary for protection also increased more among Black than White individuals in March 2021 (*b* = 0.221; *P* = .01) and April 2021 (*b* = 0.187; *P* = .04). Beliefs that the vaccines are safe and effective (*b* = 0.125; *P* < .001) and necessary (*b* = 0.405; *P* < .001) were positively associated with vaccination intention. There was no evidence that these associations varied by race.

**Conclusions and Relevance:**

This survey study suggests that the intention of Black individuals to be vaccinated was initially comparable to that of White individuals but increased more rapidly. There is some evidence that this increase is associated with changes in beliefs about the vaccine. Vaccination rates continue to be lower among Black individuals than White individuals, but these results suggest that this might be less likely the result of vaccine hesitancy than other factors.

## Key Points

**Question:**

How has COVID-19 vaccine hesitancy changed among Black and White individuals in the US since vaccines became publicly available?

**Findings:**

This survey study of 1200 US adults found that COVID-19 vaccine hesitancy decreased more rapidly among Black individuals than among White individuals since December 2020. A key factor associated with this pattern seems to be the fact that Black individuals more rapidly came to believe that vaccines were necessary to protect themselves and their communities.

**Meaning:**

This study suggests that ongoing efforts to increase vaccine uptake among Black individuals in the US should attend to a range of vaccination barriers beyond vaccine hesitancy.

## Teaser

This survey study compares changes in COVID-19 vaccine hesitancy between Black and White individuals in the US and examines mechanisms that might help explain the observed differences.

## Introduction

Across the United States, Black and African American communities have been disproportionately affected by COVID-19, with higher case rates,^1^ more deaths,^2^ and more severe economic effects^3^ than other racial and ethnic communities. Given the high infectiousness and widespread prevalence of COVID-19,^4^ vaccination is an essential tool for mitigating the pandemic and its disparate effects on marginalized communities. Highly effective vaccines are widely available across the US, but vaccination rates among Black individuals in the US are lower than those among other racial and ethnic populations.^5,6^

Two types of obstacles are associated with this gap: access and hesitancy. Access barriers, such as distant vaccine sites, lack of transportation, and inflexible work hours, impede the use of vaccines, especially within vulnerable communities.^7^ Vaccine hesitancy, defined as “delay in acceptance or refusal of vaccines despite availability,”^8^^(p4163)^ has been a challenge to infectious disease control over the past 2 decades.^9,10,11,12^

The discussion of COVID-19 vaccination rates has focused on the high rates of vaccine hesitancy in marginalized racial and ethnic communities. Recent studies indicate that individuals in the US are more hesitant to use COVID-19 vaccines than other vaccines; this pattern is particularly acute among Black individuals and members of other marginalized racial and ethnic groups.^13,14^ We argue, however, that any emphasis on hesitancy as the primary challenge to vaccination among Black individuals in the US would be a mistake. Although there is good reason to expect the level of hesitancy among this group to be higher at first, there is also reason to anticipate that Black individuals might accept COVID-19 vaccines more quickly than White individuals.

The rate of COVID-19 vaccine hesitancy increased across US populations between the spring of 2020, when the pandemic first emerged (and vaccines were only hypothetical), and December 2020, when the first COVID-19 vaccine became publicly available.^15,16^ This trend was especially pronounced among Black individuals, who consistently had the highest rates of vaccine hesitancy among US racial and ethnic groups.^17,18,19^ By December 2020, only 36% to 49% of Black individuals (compared with 44%-59% of White individuals) intended to be vaccinated when they became eligible.^13,15,16,20^ COVID-19 vaccine hesitancy (among all groups) is primarily attributed to concerns about safety, effectiveness, and adverse effects,^13,17,18,21,22^ although it has also been associated with virus-related conspiracy beliefs.^23,24^ The rate of vaccine hesitancy in December 2020 was higher among Black individuals than among White individuals even after controlling for perceptions that vaccines are important, safe, and effective.^17,25^

Among Black individuals in the US, historical racism and institutional racism also drive vaccine distrust. Historical traumas, such as the Tuskegee syphilis study and the unethical and nonconsensual use of cancer cells from Henrietta Lacks, provide important context for understanding vaccine hesitancy among Black individuals.^13,26,27,28^ Everyday exposure to institutionalized racism in health care is equally important, cultivating distrust of health care professionals and researchers.^29,30^ Black individuals in the US recognize that members of their communities often experience neglect and inadequate treatment in health care settings and that these factors contribute to worse health outcomes. Together these factors are associated with Black individuals’ hesitancy to getting vaccinated against COVID-19.

There are countervailing forces, however, that suggest that the attitudes of Black individuals toward COVID-19 vaccination could change rapidly. Alongside deep-seated suspicions toward medical and scientific institutions, Black individuals are also strongly motivated to protect themselves from health-related neglect and discrimination and to ensure that they have access to the information and health care necessary to avoid negative health outcomes and community-wide health disparities. The determination of Black individuals in the US to protect themselves, their families, and their communities is reflected in widespread efforts to provide community-specific health education, identify trustworthy health partners, and cultivate trust in partners’ advice.^7,31^ Although the desire among Black individuals to protect themselves and their communities from poor health outcomes might initially be associated with suspicion of vaccines considered new or experimental,^19^ the same desire can motivate Black community members to get vaccinated once vaccines are believed to be safe, effective, and necessary.^13^

We collected panel data, tracking the same individuals over 7 months to compare changes in vaccine hesitancy between Black and White individuals in the US and assessing the potential mechanisms that could help explain these changes. We formulated the hypotheses explored in this study prior to analysis but after data collection began. We hypothesized that Black individuals would exhibit a lower willingness to get vaccinated when COVID-19 vaccines first became available but that their willingness to get vaccinated would increase more rapidly over time compared with White individuals. We also considered possible explanations for differential changes in vaccine willingness across racial groups. We anticipated that Black individuals would more quickly come to believe that COVID-19 vaccines are necessary to protect themselves and their communities and that vaccines are safe and effective. We further hypothesized that these beliefs would help explain vaccine intentions. Finally, we hypothesized that believing that COVID-19 vaccination is a necessary form of protection and believing that it is safe and effective would both be more strongly associated with vaccine intention among Black individuals than White individuals.

## Methods

### Data Collection

This study is part of a larger project examining attitudes, beliefs, and misperceptions about COVID-19 among individuals in the US. The market research firm YouGov conducted a 7-wave online panel study with a representative sample of adults in the US, contacting participants monthly from December 2020 to June 2021. YouGov uses a sample-matching method to construct representative samples from a large online opt-in panel. The company draws a random hypothetical sample from census data (specifically, the 2018 American Community Survey 1-year sample^32^) corresponding to the target population (the sampling frame) and selects panelists who match sampling frame members’ demographic characteristics. The matched cases are then weighted to the sampling frame using propensity scores. YouGov provides poststratification weights, based on 2016 US presidential vote choice, age, race and ethnicity, and educational level. Survey results are reported in accordance with American Association for Public Opinion Research (AAPOR) guidelines for nonprobability internet panels.^33^ This study received approval from The Ohio State University institutional review board. All participants provided informed consent via an electronic form before completing the study. Participants were compensated by YouGov for their participation.

### Outcome Measures

Survey questions were developed by reviewing previously used questions on vaccination and on COVID-19 vaccination specifically, including survey items from the Centers for Disease Control and Prevention (CDC) Community Survey Question Bank, the Societal Experts Action Network (SEAN) COVID-19 Survey Archive, and the University of Southern California Understanding Coronavirus in America panel surveys (see eAppendix in the Supplement for question wording). Vaccine intention, a measure of behavioral intention, was measured on a 6-point scale (baseline mean [SD] score, 4.09 [1.86]), where 1 indicated extremely unlikely and 6 indicated extremely likely. Beginning in April, we added an item asking participants whether they had received at least 1 dose of the COVID-19 vaccine (dichotomous; yes or no). Individuals who said they had already been vaccinated were coded as “extremely likely” to get vaccinated. For descriptive purposes, we also recoded the vaccine intention measure to compute a dichotomous measure of vaccine hesitancy, which indicated that a participant was unlikely or extremely unlikely to get vaccinated (Table).

**Table.** Sample Demographic Characteristics

Characteristic^a^	Unweighted No. at baseline (weighted % of sample at baseline) (N = 1200)
Vaccine hesitancy at baseline^b^	
Overall	294 (29.4)
Among Black, non-Hispanic participants	33 (37.9)
Among White, non-Hispanic participants	219 (28.2)
Race and ethnicity	
Non-Hispanic	
Black	107 (12.2)
White	921 (64.0)
Other^c^	76 (5.7)
Hispanic	63 (15.0)
≥2 Races, non-Hispanic	32 (3.0)
Age, y	
18-29	102 (17.5)
30-44	285 (24.7)
45-59	314 (22.6)
≥60	499 (35.3)
Sex	
Male	507 (48.0)
Female	693 (52.0)
Educational level	
Less than high school	29 (4.2)
High school	291 (32.6)
Some college	446 (32.6)
Bachelor’s degree or higher	434 (30.6)
Geographical location	
Northeast	206 (17.6)
Midwest	285 (20.3)
South	443 (39.0)
West	266 (23.1)
Political party affiliation	
Democrat	615 (45.0)
Independent or other	190 (16.4)
Republican	395 (38.6)

^a^ All demographic characteristics, including race, were self-reported by survey respondents.

^b^ Participants who say they are unlikely or extremely unlikely to get vaccinated.

^c^ Choices were Asian, Native American, Middle Eastern, or other.

These analyses also used measures of 4 beliefs about COVID-19 vaccines: that they are safe, effective, necessary to protect the health of the community, and necessary to protect oneself. (These questions asked whether vaccines “will be” safe, effective, and so on, at baseline because vaccines were not available to the public at that time). The 4 items were presented in a grid, and responses were measured on a 5-point scale anchored by “strongly disagree” and “strongly agree” (with higher scores denoting greater agreement). We constructed 2 mean values: the belief that the vaccines are safe and effective (*r* > .83 in all waves; baseline mean [SD] score, 3.55 [1.01]) and the belief that they provide needed protection for oneself and one’s community (*r* > .86 in all waves; baseline mean [SD] score, 3.74 [1.23]). These mean values allowed us to assess the outcome of the belief that vaccines are safe and effective—the most commonly presumed mechanisms of vaccine intention—as well as the outcome of belief in the necessity of vaccines for protection. Analyses treating beliefs about safety and effectiveness separately yielded similar results (eFigures 10-14 in the Supplement).

### Statistical Analysis

To test whether the rate of change in vaccination intention differed by race, we estimated a fixed-effects regression model. The model included a factor variable corresponding to the survey wave as well as interaction terms between each wave indicator and several demographic characteristics that might be associated with different rates of change over time, including dummy variables for race (Black), political party affiliation (Democrat or Republican), and sex (male). The model did not include controls for stable individual-level differences because the fixed-effects approach effectively controls for these factors.^34^ Survey weights were applied when computing descriptive statistics but not in inference tests, given our use of fixed-effects regression.

Statistical analyses were conducted from April 1 to July 1, 2021, using Stata/BE, version 17.0 (StataCorp LLC) and the coefplot (Ben Jann), admetan (David Fisher), and gwtmean (David Kantor) packages. All hypotheses were directional and tested with 2-sided *P* values, with *P* < .05 considered statistically significant. We used listwise deletion to handle missing data. To ensure that our results were not the product of attrition, we replicated all analyses, including only participants who completed all 7 waves. All associations were of comparable magnitude and direction when analyzed with the more restrictive sample (eFigures 4-9 in the Supplement).

## Results

The participation rate for the nonprobability internet panel was 57.0% (1264 of 2218). There were 1200 participants in the baseline survey, with a weighted mean (SD) age of 49.5 (17.6) years, among whom 693 (52.0%; weighted) were women, 107 (12.2%; weighted) were non-Hispanic Black individuals, and 921 (64.0%; weighted) were non-Hispanic White individuals. Retention rates were high in subsequent waves. There were 984 participants (82.0% retention) in wave 2, 892 (90.7%) in wave 3, 791 (88.7%) in wave 4, 736 (93.0%) in wave 5, 674 (91.6%) in wave 6, and 620 (92.0%) in wave 7. Approximately half (51.7% [620]) of the sample completed all 7 waves. Only non-Hispanic Black and White participants were included in the analyses reported here (n = 1028). The majority of the sample was White (n = 921), but the sample included 107 Black participants. There was no evidence of disproportionate attrition among Black respondents. Other demographic characteristics were comparable to the general population, although the sample did exhibit modestly higher educational attainment than the national average (Table).

**Figure 1.**
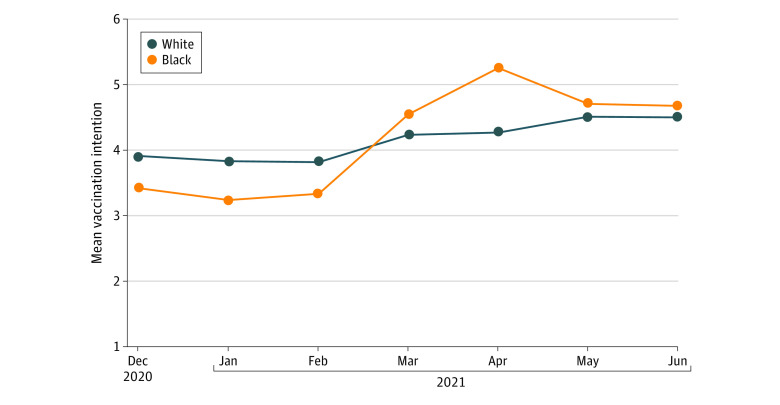
Mean Vaccination Intention Over Time by Race Weighted data in which intention is measured on a 6-point scale (where 1 indicates extremely unlikely and 6 indicates extremely likely).

Visual inspection of mean scores over time suggests that Black individuals had weaker vaccination intentions than White individuals in December 2020 (Figure 1), but the mean (SD) difference was not statistically significant (White participants, 3.927 [1.981]; Black participants, 3.437 [1.513]; *P* = .07). Although hesitance rates in the 2 groups were not statistically different at baseline in this study, the lower raw mean for Black than for White participants is consistent with other data collected at the time.^13,15,16,20^

Visual inspection further suggests that this association changed, such that the vaccine intention level of Black participants surpassed that of White participants by March 2021. Our data show that the vaccination intentions of Black participants peaked in April 2021, followed by a slight decrease in the months that followed. From these data, we cannot know the reason for the decrease in May 2021, but it might be associated with the highly publicized 10-day pause in US Food and Drug Administration and CDC authorization for the Johnson & Johnson (Janssen) COVID-19 vaccine that occurred in late April. This speculation is not related to our interpretation of the results of this study.

**Figure 2.**
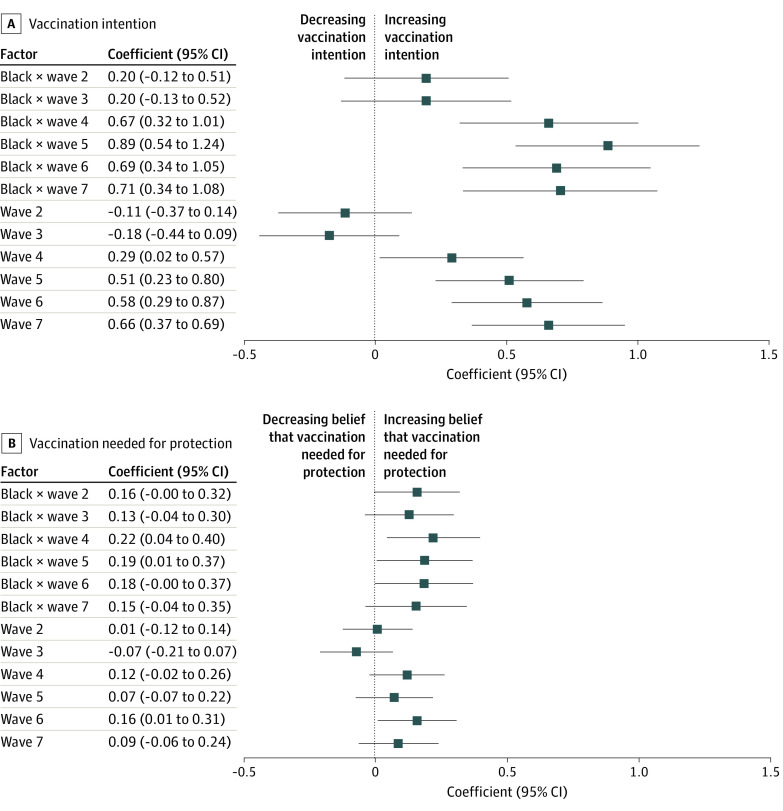
Association of Race With Vaccination Intention and Vaccine Beliefs Over Time Estimated using a fixed-effect regression model. Comparison groups are wave 1 and White individuals in the US.

The fixed-effect regression models, which provide a more robust test of our hypotheses, show that the interaction between race and wave was significant for the last 4 waves (Figure 2A; eTable 1 in the Supplement). Compared with baseline, Black participants’ vaccine intention had increased more than that of White participants in wave 4 (March 2021; *b* = 0.666; *P* < .001), wave 5 (April 2021; *b* = 0.890; *P* < .001), wave 6 (May 2021; *b* = 0.695; *P* < .001), and wave 7 (June 2021; *b* = 0.709; *P* < .001).

Next, we examined 2 factors that could be associated with more rapid increases in Black participants’ willingness to get vaccinated for COVID-19: belief that the vaccines are safe and effective and belief that the vaccines are needed for protecting oneself and one’s community. Visual inspection of the weighted mean values over time suggests that only beliefs about the vaccines’ role in providing protection changed at a rate that differs by race (eFigures 1 and 2 in the Supplement). A fixed-effects regression model confirmed that change over time in the perceived protective value of the vaccines was contingent on race. Compared with baseline, Black participants’ perceptions improved more than those of White participants in wave 4 (*b* = 0.221; *P* = .01) and wave 5 (*b* = 0.187; *P* = .04) (Figure 2B; eTable 2 in the Supplement). We found no evidence that changes in beliefs about the vaccines’ safety and effectiveness were associated with race (eFigure 3 in the Supplement).

**Figure 3.**
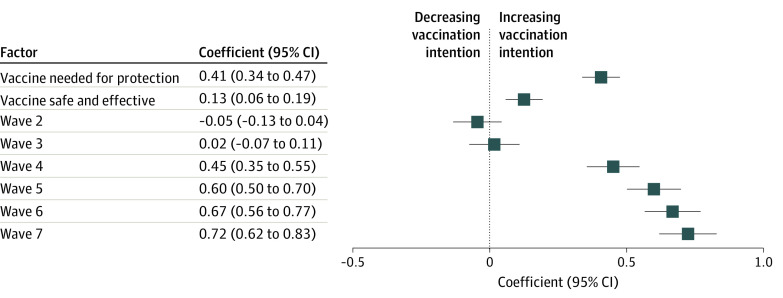
Association of Vaccine Beliefs With Vaccination Intention Estimated using a fixed-effects regression model.

Finally, we tested the association between the 2 sets of vaccine beliefs—that COVID-19 vaccines are safe and effective and that COVID-19 vaccines are necessary for the protection of oneself and one’s community—and the intention to get vaccinated. We again estimated 2 fixed-effects regression models assessing vaccine intention. One included only the 2 belief factors and wave indicators; the other added the interactions of each belief factor with race. Both vaccine beliefs were positively associated with vaccination intention (the vaccine provides needed protection: *b* = 0.405; *P* < .001; the vaccine is safe and effective: *b* = 0.125; *P* < .001) (Figure 3; eTable 3 in the Supplement), but there was no evidence that the association between vaccination intention and either the protection beliefs or the safety and effectiveness beliefs was contingent on race. Finally participants’ intention to vaccinate was significantly higher in waves 4 through 7 compared with their intentions in the baseline wave.

## Discussion

The findings support our central hypothesis that, while Black and White individuals in the US were comparably hesitant to get vaccinated when the COVID-19 vaccines first became available, Black individuals more quickly overcame this hesitancy than White individuals. Observing the same individuals over 7 months also provided modest evidence for a mechanism that could help explain differential increases in vaccination intentions by race. Individuals in the US became more likely to believe that COVID-19 vaccines are safe, effective, and necessary to protect oneself and one’s community over time, and these beliefs were all positively associated with personal intent to get vaccinated. Black individuals experienced a more rapid increase in their belief that COVID-19 vaccination is necessary for protection than White individuals. This moderating association appears to help to explain the more rapid increase in vaccination intentions among Black individuals. We found no evidence, however, that believing COVID-19 vaccines are necessary for protection varies by race. That is, although changes in beliefs about the vaccines’ protective role are contingent on race, the association of these beliefs with vaccine intention is not.

The extant literature offers ample evidence that vaccine hesitancy in Black communities is due at least in part to institutional mistrust, which is itself grounded in historical and contemporary experiences of racism.^26,27,29,30^ Our findings suggest that hesitancy is malleable, at least in the context of COVID-19. Black individuals in the US are cautious in their use of novel medical technologies for good reason—the history of abuse by medical and research communities is real—but they are just as likely as White individuals to embrace vaccination once they are convinced that vaccines are safe, effective, and necessary. Vaccine mistrust grounded in community experiences of racism might therefore be characterized not as resistance to protective health behavior but instead as an expression of commitment to protective health behavior. Our results suggest that messages about the protection that COVID-19 vaccines can confer on individuals and communities might resonate especially strongly with Black audiences. This dynamic likely results from the purposeful efforts of Black leaders and community educators, who have routinely included messages about protective and community impact in the context of consistent and proactive efforts to disseminate information about COVID-19 vaccines.^35,36,37,38,39^ If so, scholars, journalists, public health advocates, and government officials engaged in vaccine education might do well to continue to deploy protection-relevant messages, partner with trusted messengers from within Black communities, and presume that community members have genuine concerns for protecting their own health and that of their communities.^40,41,42,43^

Despite encouraging shifts in hesitancy among Black individuals in the US, current surveillance data indicate that vaccine uptake rates among Black individuals still trail those among White individuals. Data collected by the CDC from the 40 states that report reliable race and ethnicity information through May 2021 indicate that the percentage of White individuals who have received at least 1 vaccine dose is approximately 1.5 times the percentage for Black individuals; the vaccination rate among White individuals is higher within every state, although the size of the differences varies.^6^ This disparity might result in part from differences in the age distributions of racial and ethnic subpopulations; fewer Black individuals than White individuals in the US are 65 years of age or older,^44^ so fewer Black individuals were eligible to be vaccinated during the age-based stages of vaccine rollout early in 2021. If this factor were the sole origin of the disparity, however, we would expect the observed discrepancy to shrink as the age eligibility widened. This has not occurred; CDC data indicate that the difference between the percentage of White individuals and the percentage of Black individuals in the US who have received at least 1 vaccine dose had increased from 7.2% as of April 1 to 8.8% as of June 1.^5^

These figures, along with our own findings, underscore the need to ensure that research and practical efforts focus on the access barriers faced by those willing to be vaccinated.^7,45,46^ Kaiser Family Foundation surveys document the substantial proportions of Black individuals who worry about such access barriers; 55% of Black individuals (vs 41% of White individuals) are very or somewhat concerned that they might miss work if the vaccine makes them sick, 37% of Black individuals (vs 24% of White individuals) worry that they might have to pay for the vaccine, 23% of Black individuals (vs 16% of White individuals) are concerned about taking time off work to get vaccinated, and 17% of Black individuals (vs 9% of White individuals) worry about finding transportation to the vaccination site.^47^

### Limitations

This study has some limitations. First, although we consider the diversity of the study sample to be a strength, it is not perfect. Its size is modest, and the sample is somewhat better educated than the US population at large. We have no reason to expect the effect of race to be contingent on educational level, but it is possible that the patterns observed here would not be replicated with a less-educated sample of participants. Second, the primary outcome studied here is not vaccine use but self-reported intention to get vaccinated; behavioral intentions are strongly but not perfectly associated with vaccination uptake.^12^ Future studies could also explore whether other vaccine-related beliefs—beyond safety, effectiveness, and need for protection—might also be associated with vaccine intention. In addition, future studies could explore how vaccination intentions are associated with other factors, such as COVID-19 diagnoses or economic effects within one’s community or family. It will also be important to study changing vaccination intentions among other marginalized racial and ethnic groups.

## Conclusions

Using 7 waves of nationally representative panel data collected between December 2020 and June 2021, this study found rapid reductions in COVID-19 vaccine hesitancy among Black individuals in the US. Although Black and White individuals entered the period of vaccine availability with a comparable willingness to get vaccinated, Black individuals overcame their hesitancy more quickly. A key factor associated with this pattern seems to be the fact that Black individuals more rapidly came to believe that vaccines were necessary to protect themselves and their communities. This research underscores the importance of ongoing research and practical efforts to ameliorate a range of barriers to receiving the COVID-19 vaccine.

## Notes

Supplement. **eAppendix.** Question Wording **eFigure 1.** Average Belief That Vaccines Are Safe and Effective Over Time, by Race **eFigure 2.** Average Belief That Vaccines Provide Needed Protection, by Race **eFigure 3.** Factors Associated With Belief That Vaccines Are Safe and Effective **eFigure 4.** Average Vaccination Intention Over Time, by Race (No Dropouts) **eFigure 5.** Association of Race With Vaccination Intention and Vaccine Beliefs, Over Time (No Dropouts) **eFigure 6.** Association of Vaccine Beliefs With Vaccination Intention (No Dropouts) **eFigure 7.** Average Belief That Vaccines Are Safe and Effective Over Time, by Race (No Dropouts) **eFigure 8.** Average Belief That Vaccines Provide Needed Protection, by Race (No Dropouts) **eFigure 9.** Factors Associated With Belief That Vaccines Are Safe and Effective (No Dropouts) **eFigure 10.** Average Belief That Vaccines Are Safe (Only) **eFigure 11.** Average Belief That Vaccines Are Effective (Only) **eFigure 12.** Factors Associated With Belief That Vaccines Are Safe (Only) **eFigure 13.** Factors Associated With Belief That Vaccines Are Effective (Only) **eFigure 14.** Association of Vaccine Beliefs (Safe and Effective Separately) With Vaccination Intention **eTable 1.** Coefficients for Fixed-Effects Regression Model of Vaccine Intention **eTable 2.** Coefficients for Fixed-Effects Regression Model of Vaccine Protection Beliefs **eTable 3.** Coefficients for Fixed-Effects Regression Model of Vaccine Intention by Vaccine Beliefs

## References

[1] Abedi V, Olulana O, Avula V, . Racial, economic and health inequality and COVID-19 infection in the United States. medRxiv. Preprint posted online May 1, 2020. doi:10.1101/2020.04.26.20079756 PMC7462354 32875535

[2] Gross CP, Essien UR, Pasha S, Gross JR, Wang SY, Nunez-Smith M. Racial and ethnic disparities in population-level COVID-19 mortality. J Gen Intern Med. 2020;35(10):3097-3099. doi:10.1007/s11606-020-06081-w 32754782 PMC7402388

[3] Hardy BL, Logan TD. Racial economic inequality amid the COVID-19 crisis. The Hamilton Project. Published August 2020. Accessed December 10, 2021. https://www.hamiltonproject.org/assets/files/EA_HardyLogan_LO_8.12.pdf

[4] World Health Organization. WHO coronavirus (COVID-19) dashboard. Accessed July 14, 2021. https://covid19.who.int/

[5] Centers for Disease Control and Prevention. COVID data tracker. Published March 28, 2020. Accessed July 14, 2021. https://covid.cdc.gov/covid-data-tracker

[6] Ndugga N, Pham O, Hill L, Artiga S, Parker N. Latest data on COVID-19 vaccinations by race/ethnicity. Kaiser Family Foundation. Published July 8, 2021. Accessed July 14, 2021. https://web.archive.org/web/20210709203754/https://www.kff.org/coronavirus-covid-19/issue-brief/latest-data-on-covid-19-vaccinations-race-ethnicity/

[7] Nemeth JM, Padamsee, TJ. Ohio’s COVID-19 populations needs assessment. College of Public Health, The Ohio State University. Published October 2020. Accessed July 14, 2021. https://cph.osu.edu/inequitable-burdens-covid-19

[8] MacDonald NE; SAGE Working Group on Vaccine Hesitancy. Vaccine hesitancy: definition, scope and determinants. Vaccine. 2015;33(34):4161-4164. doi:10.1016/j.vaccine.2015.04.036 25896383

[9] Larson HJ, Cooper LZ, Eskola J, Katz SL, Ratzan S. Addressing the vaccine confidence gap. Lancet. 2011;378(9790):526-535. doi:10.1016/S0140-6736(11)60678-8 21664679

[10] Black S, Rappuoli R. A crisis of public confidence in vaccines. Sci Transl Med. 2010;2(61):mr1. doi:10.1126/scitranslmed.3001738 21148125

[11] Olive JK, Hotez PJ, Damania A, Nolan MS. The state of the antivaccine movement in the United States: a focused examination of nonmedical exemptions in states and counties. PLoS Med. 2018;15(6):e1002578. doi:10.1371/journal.pmed.1002578 29894470 PMC5997312

[12] Quinn SC, Jamison AM, An J, Hancock GR, Freimuth VS. Measuring vaccine hesitancy, confidence, trust and flu vaccine uptake: results of a national survey of White and African American adults. Vaccine. 2019;37(9):1168-1173. doi:10.1016/j.vaccine.2019.01.033 30709722

[13] National Foundation for Infectious Diseases. National survey: Black adult perspectives on COVID-19 and flu vaccines. Published February 4, 2021. Accessed July 14, 2021. https://www.nfid.org/national-survey-black-adult-perspectives-on-covid-19-and-flu-vaccines/

[14] Webb Hooper M, Nápoles AM, Pérez-Stable EJ. No populations left behind: vaccine hesitancy and equitable diffusion of effective COVID-19 vaccines. J Gen Intern Med. 2021;36(7):2130-2133. doi:10.1007/s11606-021-06698-5 33754319 PMC7985226

[15] Szilagyi PG, Thomas K, Shah MD, . National trends in the US public’s likelihood of getting a COVID-19 vaccine—April 1 to December 8, 2020. JAMA. 2020;325(4):396-398. doi:10.1001/jama.2020.26419 33372943 PMC7772743

[16] Nguyen KH, Srivastav A, Razzaghi H, . COVID-19 vaccination intent, perceptions, and reasons for not vaccinating among groups prioritized for early vaccination—United States, September and December 2020. MMWR Morb Mortal Wkly Rep. 2021;70(6):217-222. doi:10.15585/mmwr.mm7006e3 33571174 PMC7877585

[17] Callaghan T, Moghtaderi A, Lueck JA, . Correlates and disparities of intention to vaccinate against COVID-19. Soc Sci Med. 2021;272:113638. doi:10.1016/j.socscimed.2020.113638 33414032 PMC7834845

[18] Ruiz JB, Bell RA. Predictors of intention to vaccinate against COVID-19: results of a nationwide survey. Vaccine. 2021;39(7):1080-1086. doi:10.1016/j.vaccine.2021.01.010 33461833 PMC7794597

[19] Fisher KA, Bloomstone SJ, Walder J, Crawford S, Fouayzi H, Mazor KM. Attitudes toward a potential SARS-CoV-2 vaccine: a survey of U.S. adults. Ann Intern Med. 2020;173(12):964-973. doi:10.7326/M20-3569 32886525 PMC7505019

[20] Stern MF, Piasecki AM, Strick LB, . Willingness to receive a COVID-19 vaccination among incarcerated or detained persons in correctional and detention facilities—four states, September-December 2020. MMWR Morb Mortal Wkly Rep. 2021;70(13):473-477. doi:10.15585/mmwr.mm7013a3 33793457 PMC8022882

[21] Kreps S, Prasad S, Brownstein JS, . Factors associated with US adults’ likelihood of accepting COVID-19 vaccination. JAMA Netw Open. 2020;3(10):e2025594. doi:10.1001/jamanetworkopen.2020.25594 33079199 PMC7576409

[22] Dodd RH, Pickles K, Nickel B, . Concerns and motivations about COVID-19 vaccination. Lancet Infect Dis. 2021;21(2):161-163. doi:10.1016/S1473-3099(20)30926-9 33338440 PMC7832277

[23] Romer D, Jamieson KH. Conspiracy theories as barriers to controlling the spread of COVID-19 in the U.S. Soc Sci Med. 2020;263:113356. doi:10.1016/j.socscimed.2020.113356 32967786 PMC7502362

[24] Pummerer L, Böhm R, Lilleholt L, Winter K, Zettler I, Sassenberg K. Conspiracy theories and their societal effects during the COVID-19 pandemic. Soc Psychol Personal Sci. Published online March 19, 2021. doi:10.1177/19485506211000217

[25] Bunch L. A tale of two crises: addressing COVID-19 vaccine hesitancy as promoting racial justice. HEC Forum. 2021;33(1-2):143-154. doi:10.1007/s10730-021-09440-0 33464452 PMC7814857

[26] Corbie-Smith G. Vaccine hesitancy is a scapegoat for structural racism. JAMA Health Forum. 2021;2(3):e210434. doi:10.1001/jamahealthforum.2021.0434 36218456

[27] Fair MA, Johnson SB. Addressing racial inequities in medicine. Science. 2021;372(6540):348-349. doi:10.1126/science.abf6738 33888630

[28] Warren RC, Forrow L, Hodge DA Sr, Truog RD. Trustworthiness before trust—COVID-19 vaccine trials and the Black community. N Engl J Med. 2020;383(22):e121. doi:10.1056/NEJMp2030033 33064382

[29] Bajaj SS, Stanford FC. Beyond Tuskegee—vaccine distrust and everyday racism. N Engl J Med. 2021;384(5):e12. doi:10.1056/NEJMpv2035827 33471971 PMC9908408

[30] Jaiswal J, Halkitis PN. Towards a more inclusive and dynamic understanding of medical mistrust informed by science. Behav Med. 2019;45(2):79-85. doi:10.1080/08964289.2019.1619511 31343962 PMC7808310

[31] Ferdinand KC. Overcoming barriers to COVID-19 vaccination in African Americans: the need for cultural humility. Am J Public Health. 2021;111(4):586-588. doi:10.2105/AJPH.2020.306135 33689446 PMC7958036

[32] U.S. Census Bureau. 2018 ACS 1-year estimates. Accessed December 12, 2021. https://www.census.gov/programs-surveys/acs/technical-documentation/table-and-geography-changes/2018/1-year.html

[33] American Association for Public Opinion Research. Standard definitions: final dispositions of case codes and outcome rates for surveys. Revised 2016. Accessed November 12, 2021. https://www.aapor.org/AAPOR_Main/media/publications/Standard-Definitions20169theditionfinal.pdf

[34] Allison PD. Liao TF, ed. *Fixed Effects Regression Models*. Vol 160. Sage Publications; 2009.

[35] African American Leadership Forum. COVID-19. Accessed July 14, 2021. https://web.archive.org/web/20210507164215/https://tcaalf.com/covid-19/

[36] American Public Health Association. COVID-19 and equity. Accessed July 14, 2021. https://apha.org/topics-and-issues/communicable-disease/coronavirus/equity

[37] The News & Observer. Rev. William Barber: “Show up and be vaccinated”. YouTube video. Accessed July 14, 2021. https://www.youtube.com/watch?v=ZLWQFsZ7Yo4

[38] The Ohio State University Wexner Medical Center. Making the decision to get the COVID-19 vaccine. Accessed July 14, 2021. https://wexnermedical.osu.edu/features/coronavirus/patient-care/covid-19-vaccine/making-the-decision-to-get-vaccinated

[39] Jones M. The COVID-19 vaccine in the Black community: let’s take care of each other. *Raleigh News & Observer*. March 16, 2021. Accessed July 14, 2021. https://www.newsobserver.com/opinion/article249951699.html

[40] Johns Hopkins Medicine. COVID-19 vaccines and people of color. Updated April 16, 2021. Accessed July 14, 2021. https://www.hopkinsmedicine.org/health/conditions-and-diseases/coronavirus/covid19-vaccines-and-people-of-color

[41] Kaiser Permanente. Supporting equitable COVID-19 vaccine education. Published March 9, 2021. Accessed July 14, 2021. https://about.kaiserpermanente.org/our-story/news/announcements/supporting-equitable-covid-19-vaccine-education

[42] Perkins DEA. A COVID-19 vaccination challenge. Am J Nurs. 2021;121(3):11-11. doi:10.1097/01.NAJ.0000737224.48167.78 33624992

[43] Privor-Dumm L, King T. Community-based strategies to engage pastors can help address vaccine hesitancy and health disparities in Black communities. J Health Commun. 2020;25(10):827-830. doi:10.1080/10810730.2021.1873463 33719889

[44] Schaeffer K. The most common age among Whites in U.S. is 58—more than double that of racial and ethnic minorities. Pew Research Center. Published July 30, 2019. Accessed July 14, 2021. https://www.pewresearch.org/fact-tank/2019/07/30/most-common-age-among-us-racial-ethnic-groups/

[45] Ward E, Halpern M, Schrag N, . Association of insurance with cancer care utilization and outcomes. CA Cancer J Clin. 2008;58(1):9-31. doi:10.3322/CA.2007.0011 18096863

[46] Simon K, Soni A, Cawley J. The impact of health insurance on preventive care and health behaviors: evidence from the first two years of the ACA Medicaid expansions. J Policy Anal Manage. 2017;36(2):390-417. doi:10.1002/pam.21972 28378959

[47] Kaiser Family Foundation. KFF COVID-19 vaccine monitor dashboard. Accessed July 14, 2021. https://www.kff.org/coronavirus-covid-19/dashboard/kff-covid-19-vaccine-monitor-dashboard/

