# Supplementary material for: Changes in COVID-19 Vaccine Hesitancy Among Black and White Individuals in the US
Source: JAMA Netw Open. 2022 Jan 21;5(1):e2144470. doi: 10.1001/jamanetworkopen.2021.44470 (PMC8783270; doi:10.1001/jamanetworkopen.2021.44470)
Supplement: Supplement. — eAppendix. Question Wording eFigure 1. Average Belief That Vaccines Are Safe and Effective Over Time, by Race eFigure 2. Average Belief That Vaccines Provide Needed Protection, by Race eFigure 3. Factors Associated With Belief That Vaccines Are Safe and Effective eFigure 4. Average Vaccination Intention Over Time, by Race (No Dropouts) eFigure 5. Association of Race With Vaccination Intention and Vaccine Beliefs, Over Time (No Dropouts) eFigure 6. Association of Vaccine Beliefs With Vaccination Intention (No Dropouts) eFigure 7. Average Belief That Vaccines Are Safe and Effective Over Time, by Race (No Dropouts) eFigure 8. Average Belief That Vaccines Provide Needed Protection, by Race (No Dropouts) eFigure 9. Factors Associated With Belief That Vaccines Are Safe and Effective (No Dropouts) eFigure 10. Average Belief That Vaccines Are Safe (Only) eFigure 11. Average Belief That Vaccines Are Effective (Only) eFigure 12. Factors Associated With Belief That Vaccines Are Safe (Only) eFigure 13. Factors Associated With Belief That Vaccines Are Effective (Only) eFigure 14. Association of Vaccine Beliefs (Safe and Effective Separately) With Vaccination Intention eTable 1. Coefficients for Fixed-Effects Regression Model of Vaccine Intention eTable 2. Coefficients for Fixed-Effects Regression Model of Vaccine Protection Beliefs eTable 3. Coefficients for Fixed-Effects Regression Model of Vaccine Intention by Vaccine Beliefs [file jamanetwopen-e2144470-s001.pdf]

Supplementary Online Content

Padamsee TJ, Bond RM, Dixon GN, et al. Changes in COVID-19 vaccine hesitancy among Black and White individuals in the US. *JAMA Netw Open.* 2022;5(1):e2144470. doi:10.1001/jamanetworkopen.2021.44470

eAppendix. Question Wording
eFigure 1. Average Belief That Vaccines Are Safe and Effective Over Time, by Race
eFigure 2. Average Belief That Vaccines Provide Needed Protection, by Race
eFigure 3. Factors Associated With Belief That Vaccines Are Safe and Effective
eFigure 4. Average Vaccination Intention Over Time, by Race (No Dropouts)
eFigure 5. Association of Race With Vaccination Intention and Vaccine Beliefs, Over Time (No Dropouts)
eFigure 6. Association of Vaccine Beliefs With Vaccination Intention (No Dropouts)
eFigure 7. Average Belief That Vaccines Are Safe and Effective Over Time, by Race (No Dropouts)
eFigure 8. Average Belief That Vaccines Provide Needed Protection, by Race (No Dropouts)
eFigure 9. Factors Associated With Belief That Vaccines Are Safe and Effective (No Dropouts)
eFigure 10. Average Belief That Vaccines Are Safe (Only)
eFigure 11. Average Belief That Vaccines Are Effective (Only)
eFigure 12. Factors Associated With Belief That Vaccines Are Safe (Only)
eFigure 13. Factors Associated With Belief That Vaccines Are Effective (Only)
eFigure 14. Association of Vaccine Beliefs (Safe and Effective Separately) With Vaccination Intention
eTable 1. Coefficients for Fixed-Effects Regression Model of Vaccine Intention
eTable 2. Coefficients for Fixed-Effects Regression Model of Vaccine Protection Beliefs
eTable 3. Coefficients for Fixed-Effects Regression Model of Vaccine Intention by Vaccine Beliefs

This supplementary material has been provided by the authors to give readers additional information about their work.

© 2022 Padamsee TJ et al. *JAMA Network Open.*

eAppendix. Question Wording

Have you received at least one dose of the COVID-19 vaccine?

1. No
2. Yes

Included starting in the fourth wave of data collection.

How likely are you to get the COVID-19 vaccine?

1. Extremely unlikely
2. Unlikely
3. Somewhat unlikely
4. Somewhat likely
5. Likely
6. Extremely likely

Only asked if participant did not indicate prior vaccination.

Please tell us how much you agree or disagree with each of the following statements about the [potential] COVID-19 vaccine. *The word "potential" was only included in the first wave of data collection.*

The COVID-19 vaccine [will be/is] safe
The COVID-19 vaccine [will be/is] effective
The COVID-19 vaccine [will be/is] necessary to protect the health of the community
The COVID-19 vaccine [will be/is] necessary to protect myself

The first wave of data collection used "will be"; all other waves used "is".

Response options

1. Strongly disagree
2. Disagree
3. Neither agree nor disagree
4. Agree
5. Strongly agree

In what year were you born?

Subtracted 2021 to determine age.

© 2022 Padamsee TJ et al. *JAMA Network Open.*

What racial or ethnic group best describes you?
1. White
2. Black or African-American
3. Hispanic or Latino
4. Asian or Asian-American
5. Native American
6. Two or more races
7. Other
8. Middle Eastern

Are you male or female?
1. Male
2. Female

What is the highest level of education you have completed?
1. Did not graduate from high school
2. High school graduate
3. Some college, but no degree (yet)
4. 2-year college degree
5. 4-year college degree
6. Postgraduate degree (MA, MBA, MD, JD, PhD, etc.)

Recoded 3 and 4 to "Some college," 5 and 6 to "Bachelor's degree or higher.

What is your State of Residence?

Recoded into four Census Bureau-designated regions.

Generally speaking, when it comes to political parties in the United States, how would you best describe yourself?
1. A strong Democrat
2. A not very strong Democrat
3. Independent, lean toward Democrat
4. Independent (close to neither party)
5. Independent, lean toward Republican
6. A not very strong Republican
7. A strong Republican
8. Something else, please specify

© 2022 Padamsee TJ et al. *JAMA Network Open.*

eFigure 1. Average Belief That Vaccines Are Safe and Effective Over Time, by Race

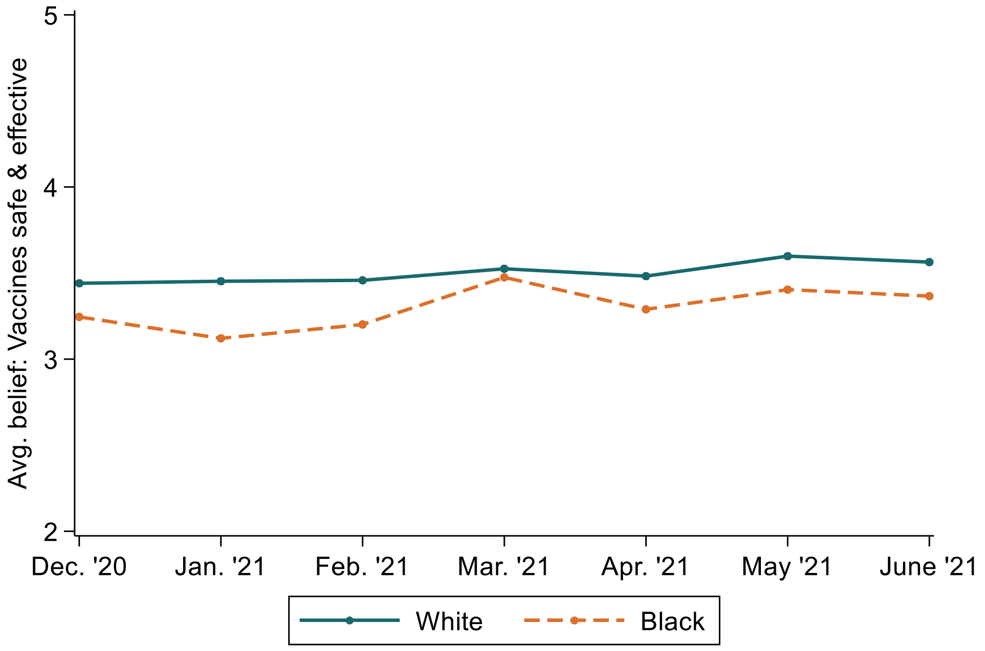

Note. Weighted data

© 2022 Padamsee TJ et al. *JAMA Network Open.*

eFigure 2. Average Belief That Vaccines Provide Needed Protection, by Race

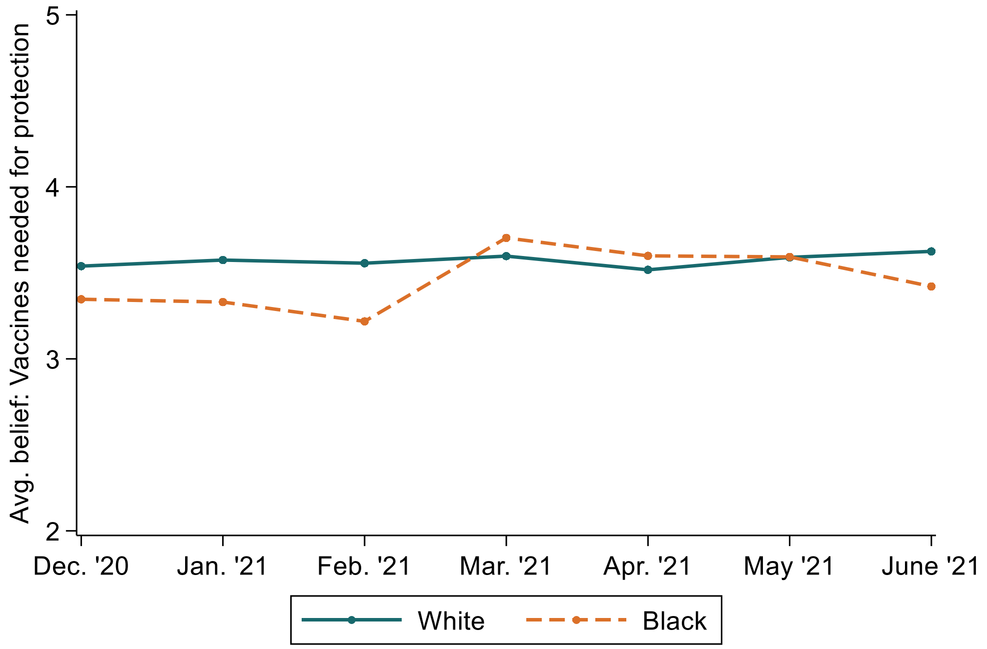

Note. Weighted data

© 2022 Padamsee TJ et al. *JAMA Network Open.*

eFigure 3. Factors Associated With Belief That Vaccines Are Safe and Effective

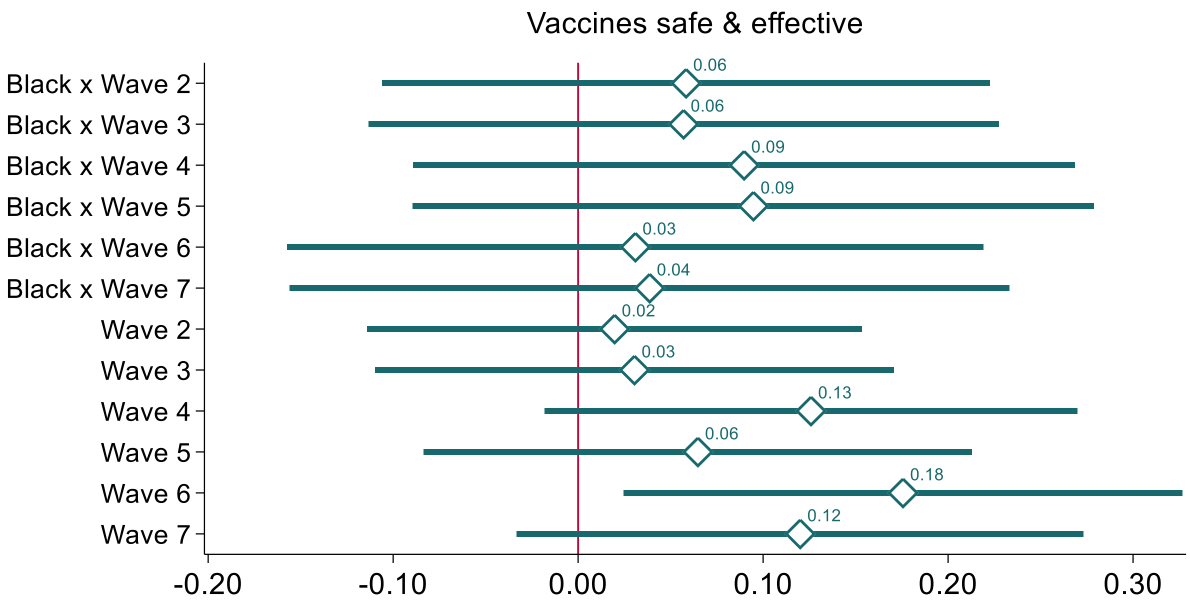

Notes. Estimated using fixed-effect regression model. Points represent coefficient estimates and horizontal lines represent 95% confidence intervals. Comparison groups are wave 1 and white.

© 2022 Padamsee TJ et al. *JAMA Network Open.*

eFigure 4. Average Vaccination Intention Over Time, by Race (No Dropouts)

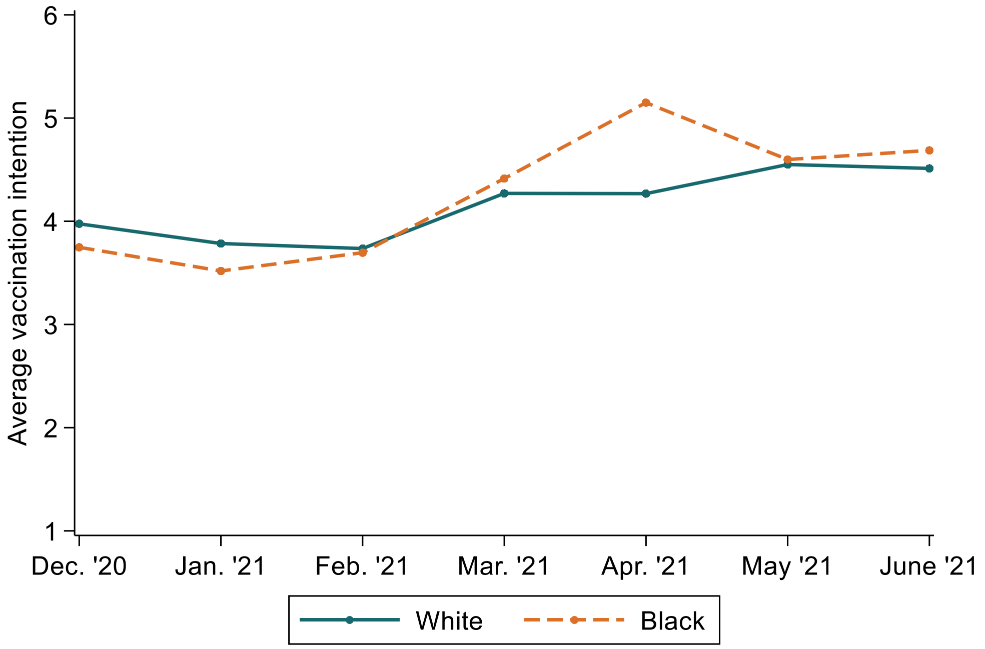

Notes. Only participants who completed all panel waves. Compare to Fig. 1.

© 2022 Padamsee TJ et al. *JAMA Network Open.*

eFigure 5. Association of Race With Vaccination Intention and Vaccine Beliefs, Over Time (No Dropouts)

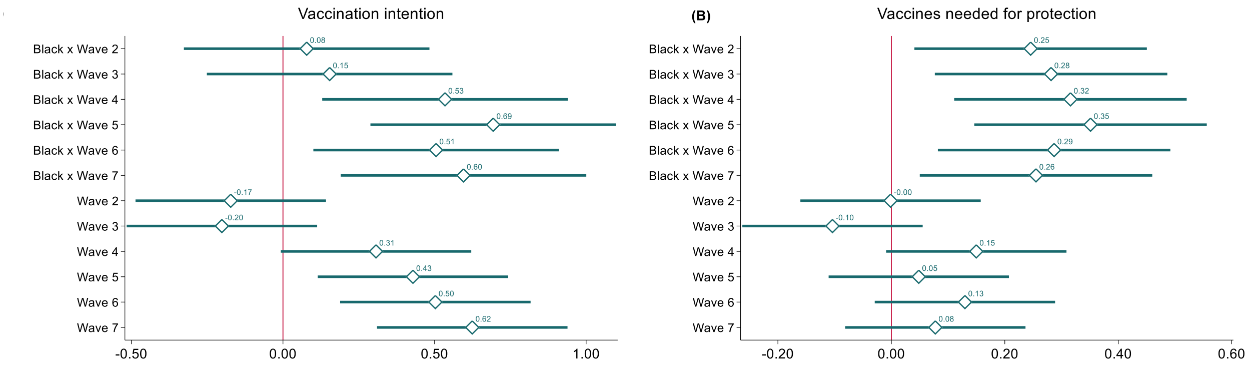

Notes. Only participants who completed all panel waves. Estimated using fixed-effect regression model. Points represent coefficient estimates and horizontal lines represent 95% confidence intervals. Comparison groups are wave 1 and white. Compare to Fig. 2.

© 2022 Padamsee TJ et al. *JAMA Network Open.*

eFigure 6. Association of Vaccine Beliefs With Vaccination Intention (No Dropouts)

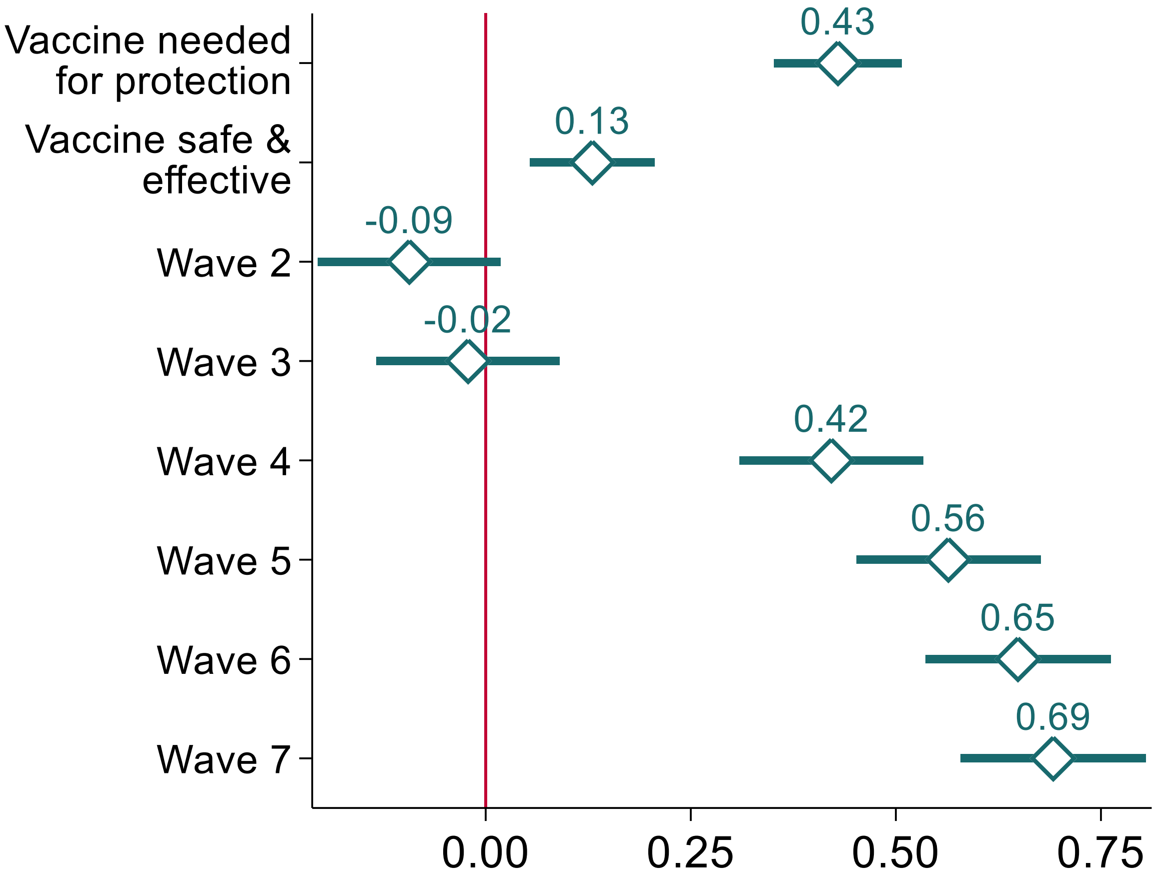

Notes. Only participants who completed all panel waves. Estimated using fixed-effects regression model. Points represent coefficient estimates and horizontal lines represent 95% confidence intervals. Compare to Fig. 3.

© 2022 Padamsee TJ et al. *JAMA Network Open.*

eFigure 7. Average Belief That Vaccines Are Safe and Effective Over Time, by Race (No Dropouts)

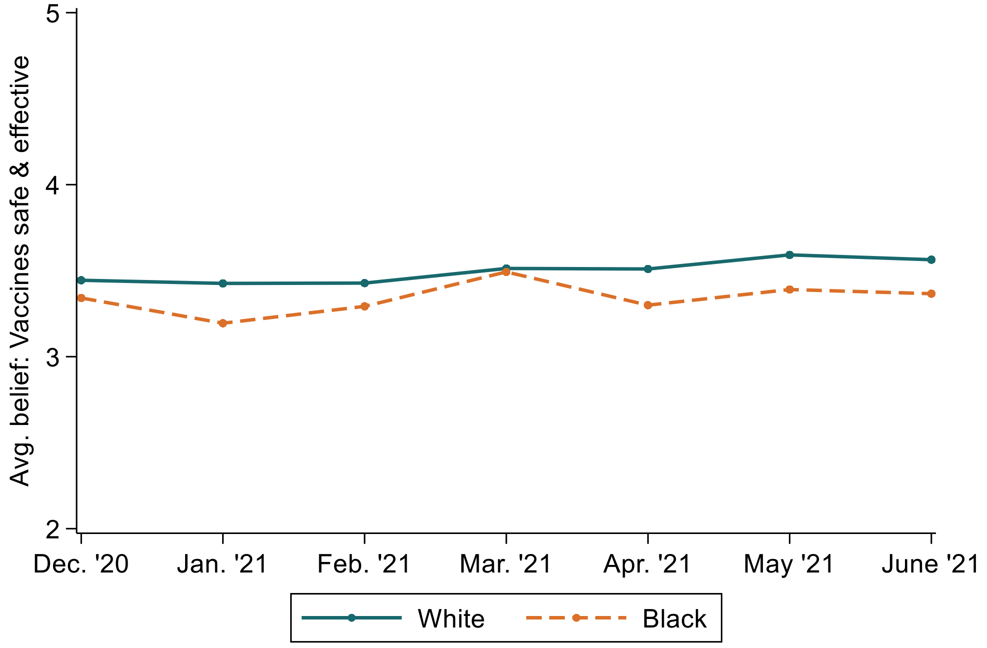

Notes. Only participants who completed all panel waves. Compare to eFig. 1.

© 2022 Padamsee TJ et al. *JAMA Network Open.*

eFigure 8. Average Belief That Vaccines Provide Needed Protection, by Race (No Dropouts)

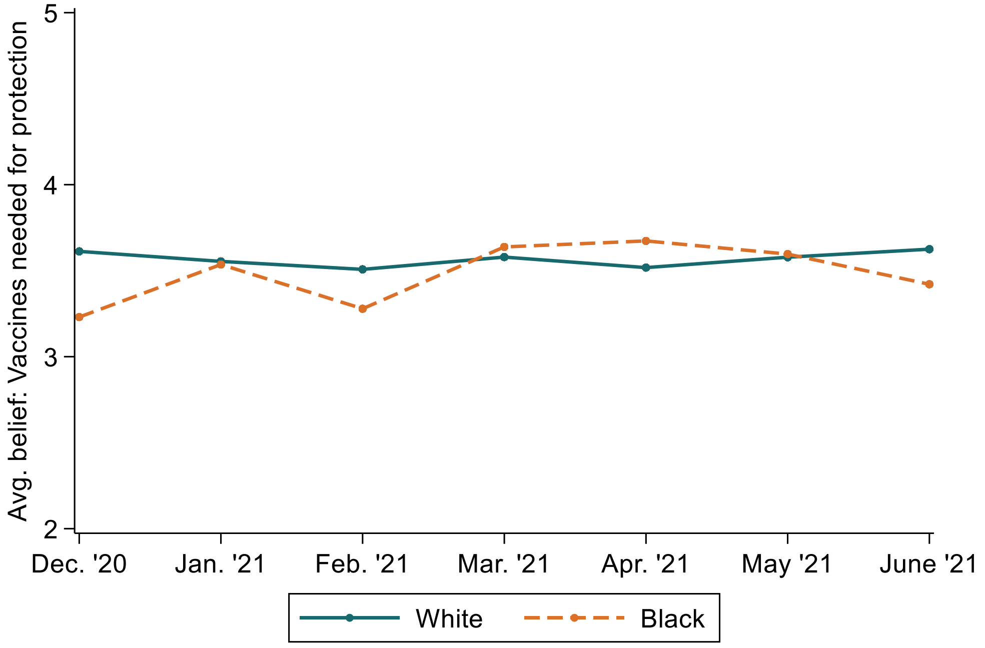

Notes. Only participants who completed all panel waves. Compare to eFig. 2.

© 2022 Padamsee TJ et al. *JAMA Network Open.*

eFigure 9. Factors Associated With Belief That Vaccines Are Safe and Effective (No Dropouts)

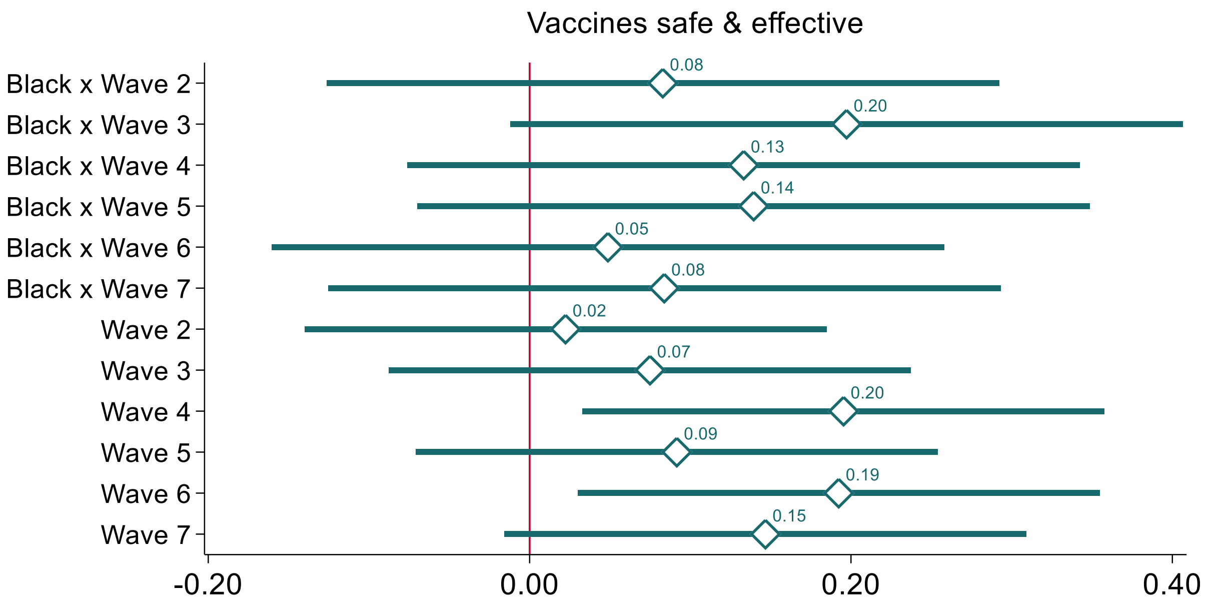

Notes. Only participants who completed all panel waves. Compare to eFig. 3.

© 2022 Padamsee TJ et al. *JAMA Network Open.*

eFigure 10. Average Belief That Vaccines Are Safe (Only)

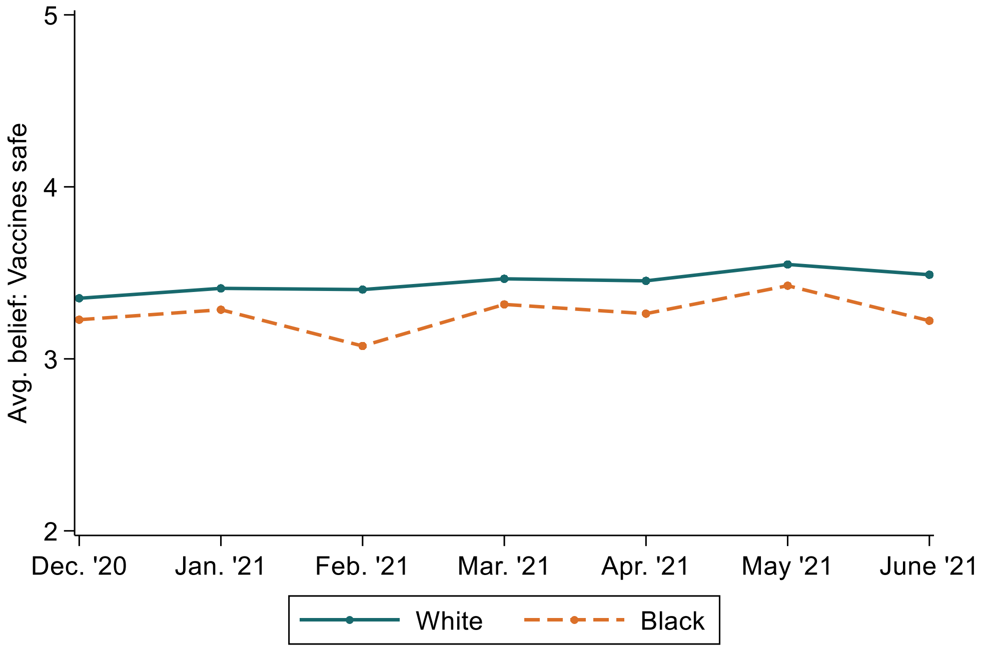

Notes. Compare to eFig. 1.

© 2022 Padamsee TJ et al. *JAMA Network Open.*

eFigure 11. Average Belief That Vaccines Are Effective (Only)

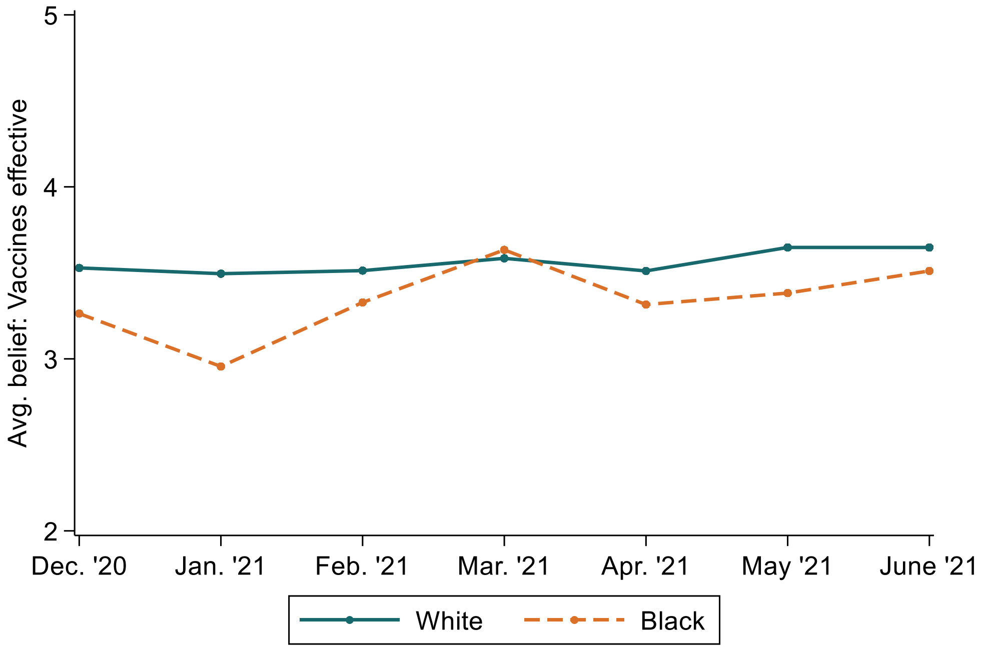

Notes. Compare to eFig. 1.

© 2022 Padamsee TJ et al. *JAMA Network Open.*

eFigure 12. Factors Associated With Belief That Vaccines Are Safe (Only)

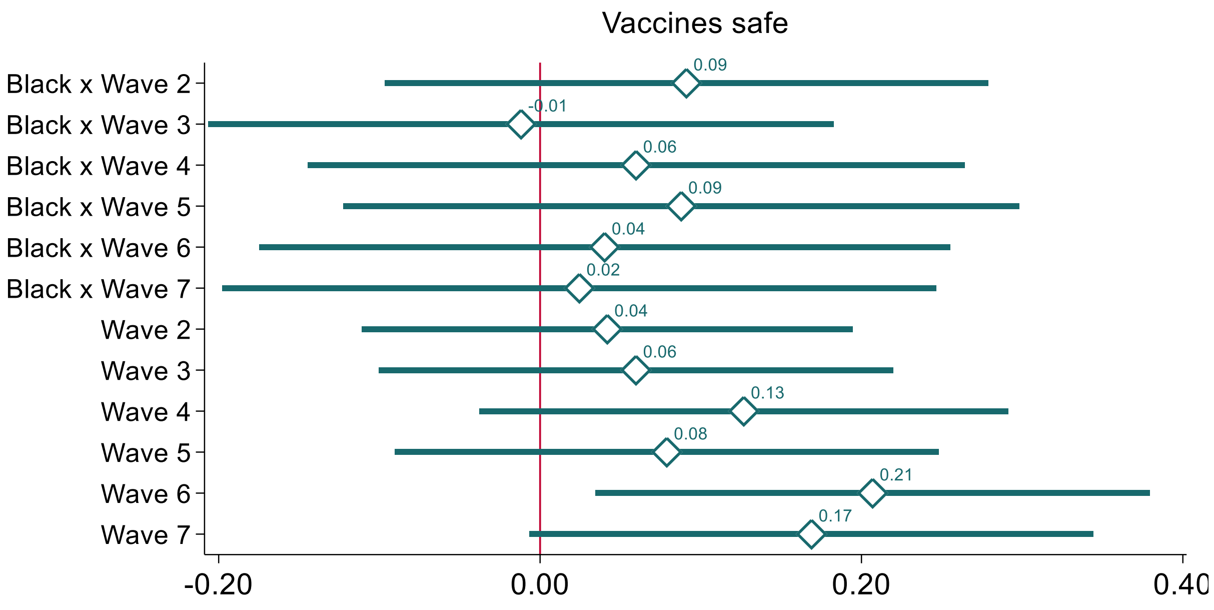

Notes. Compare to eFig. 3.

© 2022 Padamsee TJ et al. *JAMA Network Open.*

eFigure 13. Factors Associated With Belief That Vaccines Are Effective (Only)

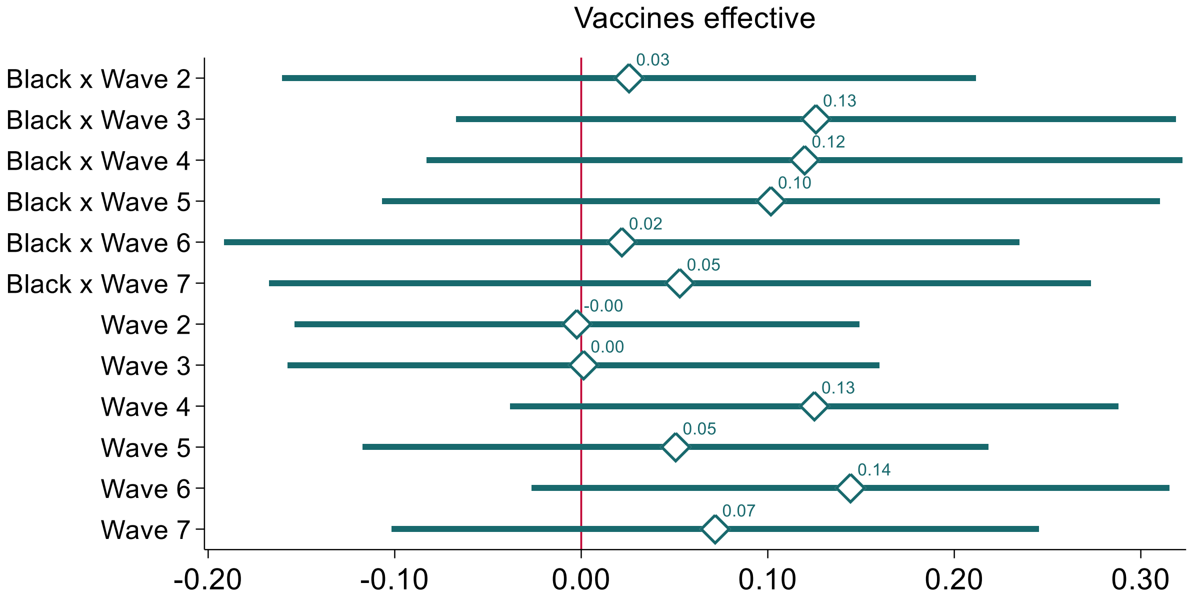

Notes. Compare to eFig. 3.

© 2022 Padamsee TJ et al. *JAMA Network Open.*

eFigure 14. Association of Vaccine Beliefs (Safe and Effective Separately) With Vaccination Intention

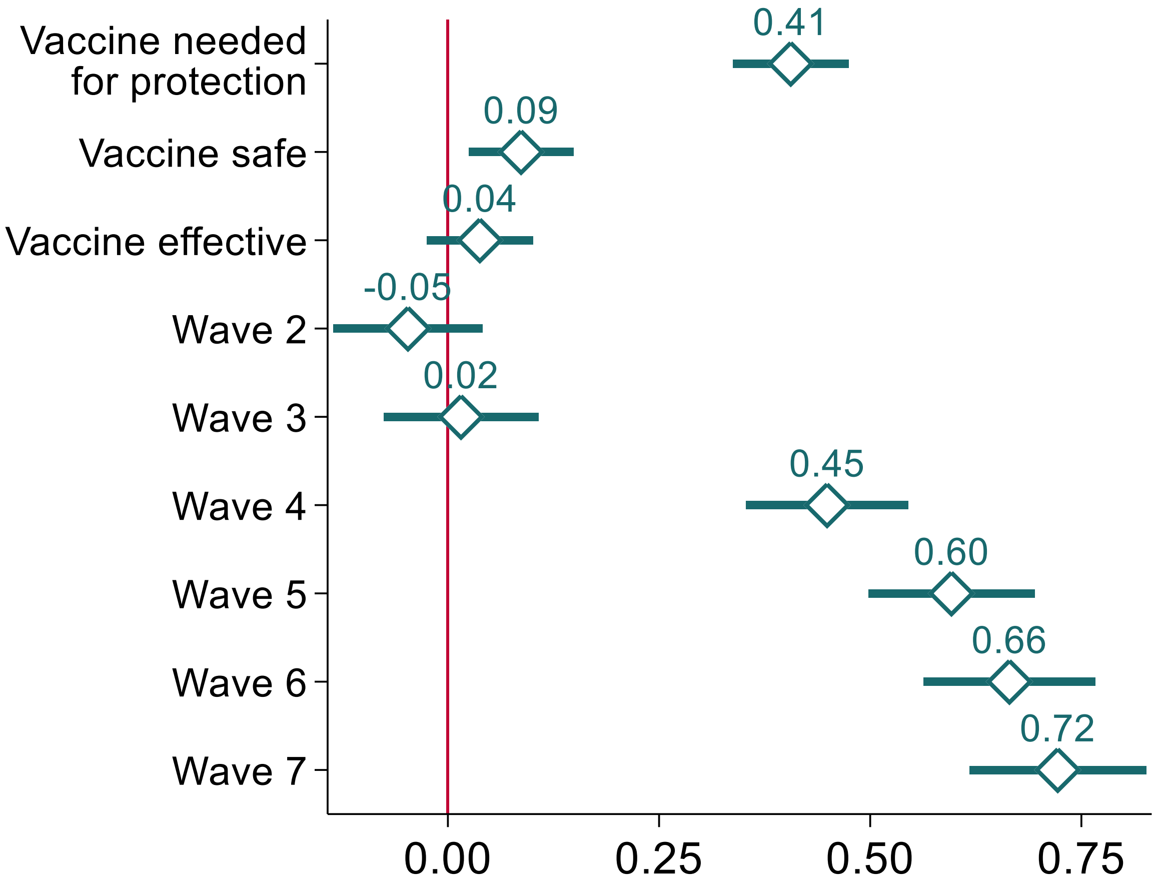

Notes. Estimated using fixed-effects regression model. Points represent coefficient estimates and horizontal lines represent 95% confidence intervals. Compare to Fig. 3.

© 2022 Padamsee TJ et al. *JAMA Network Open.*

eTable 1. Coefficients for Fixed-Effects Regression Model of Vaccine Intention

	Coefficient	Std. error	*P* value	95% CI	
Black x W2 [a]	0.197	0.160	0.219	-0.117	0.511
Black x W3	0.196	0.166	0.239	-0.130	0.521
Black x W4	0.666	0.174	0.000	0.324	1.008
Black x W5	0.890	0.179	0.000	0.538	1.242
Black x W6	0.695	0.184	0.000	0.335	1.055
Black x W7	0.709	0.190	0.000	0.337	1.080
Male x W2 [b]	0.009	0.094	0.923	-0.176	0.194
Male x W3	0.143	0.097	0.142	-0.048	0.334
Male x W4	0.021	0.101	0.834	-0.178	0.220
Male x W5	-0.010	0.104	0.924	-0.214	0.194
Male x W6	0.099	0.107	0.356	-0.111	0.309
Male x W7	-0.038	0.110	0.728	-0.254	0.177
Democrat x W2 [c]	0.112	0.137	0.414	-0.157	0.382
Democrat x W3	0.283	0.143	0.048	0.003	0.564
Democrat x W4	0.258	0.148	0.080	-0.031	0.547
Democrat x W5	0.102	0.152	0.502	-0.196	0.400
Democrat x W6	0.075	0.155	0.626	-0.228	0.379
Democrat x W7	0.120	0.157	0.445	-0.188	0.429
Republican x W2 [c]	0.037	0.145	0.797	-0.247	0.321
Republican x W3	0.009	0.151	0.955	-0.288	0.306
Republican x W4	0.056	0.156	0.718	-0.250	0.363
Republican x W5	0.054	0.160	0.734	-0.260	0.368
Republican x W6	0.085	0.164	0.603	-0.236	0.407
Republican x W7	0.136	0.167	0.417	-0.192	0.464
Wave 2 [d]	-0.115	0.130	0.379	-0.370	0.141
Wave 3	-0.175	0.137	0.200	-0.443	0.093
Wave 4	0.293	0.140	0.037	0.018	0.569
Wave 5	0.515	0.144	0.000	0.232	0.798
Wave 6	0.582	0.147	0.000	0.293	0.871
Wave 7	0.664	0.149	0.000	0.371	0.957
Intercept	4.159	0.032	0.000	4.095	4.222
Number of observations					5113
Number of groups					1028
F (30, 4055)					20.816

Note. Comparison groups are (a) white, (b) female, (c) Independent, and (d) Wave 1

© 2022 Padamsee TJ et al. *JAMA Network Open.*

eTable 2. Coefficients for Fixed-Effects Regression Model of Vaccine Protection Beliefs

	Coefficient	Std. error	*P* value	95% CI	
Black x W2 [a]	0.158	0.083	0.057	-0.005	0.321
Black x W3	0.129	0.086	0.133	-0.039	0.298
Black x W4	0.221	0.090	0.014	0.044	0.398
Black x W5	0.187	0.093	0.044	0.005	0.370
Black x W6	0.185	0.095	0.052	-0.002	0.371
Black x W7	0.155	0.098	0.115	-0.038	0.347
Male x W2 [b]	-0.034	0.049	0.488	-0.129	0.062
Male x W3	-0.047	0.050	0.348	-0.146	0.052
Male x W4	-0.071	0.053	0.174	-0.174	0.032
Male x W5	-0.007	0.054	0.894	-0.113	0.099
Male x W6	-0.093	0.055	0.094	-0.201	0.016
Male x W7	-0.084	0.057	0.140	-0.196	0.028
Democrat x W2 [c]	0.068	0.071	0.343	-0.072	0.207
Democrat x W3	0.251	0.074	0.001	0.106	0.397
Democrat x W4	0.097	0.076	0.202	-0.052	0.247
Democrat x W5	0.108	0.079	0.171	-0.046	0.262
Democrat x W6	0.101	0.080	0.206	-0.056	0.258
Democrat x W7	0.185	0.082	0.023	0.025	0.345
Republican x W2 [c]	0.007	0.075	0.931	-0.141	0.154
Republican x W3	0.087	0.078	0.268	-0.067	0.241
Republican x W4	-0.139	0.081	0.086	-0.298	0.020
Republican x W5	-0.109	0.083	0.187	-0.272	0.053
Republican x W6	-0.141	0.085	0.097	-0.308	0.025
Republican x W7	-0.039	0.087	0.651	-0.209	0.131
Wave 2 [d]	0.009	0.067	0.893	-0.123	0.141
Wave 3	-0.072	0.071	0.312	-0.210	0.067
Wave 4	0.120	0.073	0.098	-0.022	0.263
Wave 5	0.072	0.075	0.335	-0.075	0.219
Wave 6	0.159	0.076	0.037	0.009	0.308
Wave 7	0.089	0.077	0.252	-0.063	0.240
Intercept	3.797	0.017	0.000	3.764	3.830
Number of observations					5113
Number of groups					1028
F (30, 4055)					3.592

Note. Comparison groups are (a) white, (b) female, (c) Independent, and (d) Wave 1

© 2022 Padamsee TJ et al. *JAMA Network Open.*

eTable 3. Coefficients for Fixed-Effects Regression Model of Vaccine Intention by Vaccine Beliefs

	**Coefficient**	**Std. error**	***P* value**	**95% CI**	
Vaccine needed for protection	0.405	0.035	0.000	0.337	0.474
Vaccine safe & effective	0.125	0.034	0.000	0.058	0.192
Wave 2 [a]	-0.045	0.045	0.315	-0.134	0.043
Wave 3	0.017	0.047	0.721	-0.075	0.108
Wave 4	0.449	0.049	0.000	0.353	0.545
Wave 5	0.598	0.050	0.000	0.500	0.696
Wave 6	0.666	0.052	0.000	0.564	0.768
Wave 7	0.722	0.053	0.000	0.617	0.826
Intercept	2.170	0.126	0.000	1.923	2.418
Number of observations	5113				
Number of groups	1028				
F (8, 4077)	109.424				

Note. a. Comparison group is Wave 1

© 2022 Padamsee TJ et al. *JAMA Network Open.*
